# Feasibility of 16S rRNA sequencing for cerebrospinal fluid microbiome analysis in cattle with neurological disorders: a pilot study

**DOI:** 10.1007/s11259-022-09949-w

**Published:** 2022-06-27

**Authors:** Sara Ferrini, Elena Grego, Ugo Ala, Giulia Cagnotti, Flaminia Valentini, Giorgia Di Muro, Barbara Iulini, Maria Cristina Stella, Claudio Bellino, Antonio D’Angelo

**Affiliations:** 1 Department of Veterinary Sciences, Clinical section, University of Turin, Largo Paolo Braccini 2, 10095 Grugliasco, TO Italy; 2 Istituto Zooprofilattico del Piemonte Liguria e Valle d’Aosta, Turin, Italy

**Keywords:** 16S rRNA gene, Cerebrospinal fluid, Central nervous system infections, Cattle, Microbial community, Next generation sequencing

## Abstract

Bacterial infection of the central nervous system (CNS) in cattle requires prompt and adequate antimicrobial treatment. The current gold standard for antemortem etiological diagnosis is cerebrospinal fluid (CSF) culture, which often yields false negative results. CSF has long been considered a sterile district in healthy patients, but this notion has been recently challenged. For this pilot study, we used 16S rRNA gene sequencing to investigate the microbial composition of CSF of cattle presenting with CNS disorders and to compare it between subjects with CNS infections and with CNS disorders of other nature. The study sample was 10 animals: 4 presenting with CNS infectious-inflammatory diseases and 6 with other CNS disorders, based on definitive diagnosis. Since the initial round of a standard 16S rRNA PCR did not yield sufficient genetic material for sequencing in any of the samples, the protocol was modified to increase its sensitivity. Bacterial genetic material was identified in 6 animals and 2 groups were formed: an infectious inflammatory (n = 3) and a noninfectious inflammatory group (n = 3). The most frequently expressed bacterial families were *Pseudomonadaceae* (44.61%), *Moraxellaceae* (19.54%), *Mycobacteriaceae* (11.80%); the genera were *Pseudomonas* (45.42%), *Acinetobacter* (19.91%), *Mycobacterium* (12.01%). There were no detectable differences in the CSF microbial composition of the samples from the two groups. Sequencing of bacterial DNA present in the CSF was possible only after increasing PCR sensitivity. The results of 16S rRNA sequencing showed the presence of a microbial community in the CSF in cattle with neurological disorders. Further studies, in which CSF samples from healthy animals and samples from the environment are included as controls, are needed.

## Introduction

Central nervous system (CNS) infection in cattle incurs economic losses due to livestock mortality, neurological impairment, and reduction of animal performance and productivity. Neurological diseases may also be zoonotic (Clarke et al. 2019). CNS infection can be caused by bacteria, viruses, fungi, and parasites, among which bacteria are the most common cause in cattle and are characterized by a rapid course, high morbidity and mortality unless treated with antimicrobials. Some of the leading bacterial agents reported are *Escherichia coli*, *Trueperella pyogenes*, *Histophilus somni*, *Pasteurella multocida* and *Listeria monocytogenes*; mixed aerobic-anaerobic bacterial infections are reported as well (Fecteau et al. 2017).

Prompt etiological diagnosis is key to the initiation of appropriate antibiotic treatment and the adoption of control and prevention measures. This can be difficult, however, because clinical signs and blood test results are often non-specific. Cerebrospinal fluid (CSF) collection can be easily and safely performed in field conditions; it is the most direct antemortem method of diagnosing CNS disease, in which advanced diagnostic imaging in large animals is much less feasible (Nagy 2017). Infectious-inflammatory disease of the CNS can usually be distinguished by abnormal increases in protein concentration, total and differential nucleated cell counts in the CSF (Scott 2004). Bacterial culture of the CSF is currently the gold standard for etiological antemortem diagnosis of bacterial CNS infection (Benninger and Steiner 2017), but culture results may not be available for at least 24–48 h and are reported to be false negative in 44–100% of cases (Scott 2004; Stokol et al. 2009). The occurrence of false negatives may be due to previous use of antibiotics or to the presence of slow-growing and fastidious microorganisms (Leber et al. 2016; Salipante et al. 2013).

There is increasing evidence for 16S rRNA gene sequencing in human medicine as a rapid and accurate means to detect bacteria in the CSF, thus overcoming the limitations of culture-based bacterial detection (Liu et al. 2015). 16S rRNA gene is a universal gene highly conserved among bacteria and sequencing of its variable regions allows differentiation between organisms at the genus level. 16S rRNA sequencing has introduced some advantages for diagnostic microbiology laboratories such as accuracy, simplification of protocols and identification of unculturable and fastidious bacteria. Furthermore, the results are in electronic formats, easy to share between laboratories and useful for pathogenic surveillance and future epidemiological studies. However, the low taxonomic resolution, the price of sequencing and the need for bioinformatic knowledge to generate results represent potential disadvantages of this technique that must be overcome (Rizal et al. 2020). Conventional methods for bacterial identification in the CSF highlight a microbe-free CSF in healthy individuals. While the CSF circulating in the human CNS has long been considered sterile because the blood-brain barrier is thought to effectively protect against microbial invasion (Sweeney et al. 2018), reports of microorganisms detected in human brains and CSF have challenged this concept (Dominy et al. 2019; Ghose et al. 2019).

Based on these premises, we hypothesized that 16S rRNA sequencing of DNA isolated from CSF samples collected in the field from cattle with neurological diseases could lead to microorganisms identification and their taxonomic classification.

In the present study, we described the distribution of microorganisms in the CSF of two groups of cattle (patients with an infectious inflammatory neurological disease and patients with nervous disorders of other nature) exploring whether there were any detectable differences in the microbial distribution between the two groups.

## Materials and methods

### Study design

The study sample was obtained from animals referred to the Neurology Service of the Veterinary Teaching Hospital, University of Turin, because of neurological signs suggestive of a CNS disorder. All underwent general physical examination, neurologic examination by a board-certified neurologist (ADA), CSF sampling in the field and analysis within 1 h after collection, blood biochemical analysis and necropsy when possible. CSF analysis also included CSF bacterial culture.

The final diagnosis was reached based on signalment, results of the neurological examination, blood and CSF analysis, response to treatment, and postmortem histopathology when performed. Information on therapies administered before referral was recorded. Two groups were formed according to the final diagnosis expressed according to the VITAMIN D classification (Jaggy 2009). The one group was composed of patients with infectious inflammatory disease of the CNS (infectious inflammatory group) and the other included patients with CNS disorders of another nature (noninfectious inflammatory group).

This prospective pilot study was conducted in accordance with current animal welfare regulations (Directive 98/58/EC and Italian Decree Law 146/2001). Samples were collected during the routine analysis required to perform the diagnostic procedure. Written informed consent was obtained from the owners to authorize veterinary assessment and treatment of their animals.

CSF was collected in the field from the lumbosacral space, as described by Mayhew (2009). If necessary, the animal was sedated with 0.05 mg/kg xylazine (Rompun, Bayer HealthCare) administered intravenously. The sampling site (5 cm x 10 cm) was clipped, surgically prepared, and locally anesthetized with 2.5 ml of procaine hydrochloride 20 mg/ml (Procamidor, Richter Pharma Ag). The skin around the sampling site was disinfected by alternating application of povidone iodine and alcohol moistened gauze three times.

Disposable sterile spinal needles (20G 0.9 mm x 90 mm or 21G 0.8 mm x 50 mm, Terumo) were used depending on the size of the animal. After correct positioning of the needle, the sample was obtained by gentle aspiration with a sterile syringe. The CSF sample was then divided into two aliquots and placed in empty sterile tubes. The tube cap was previously sterilized with alcohol and a new sterile hypodermic needle was used to transfer the CSF into each tube. The first aliquot, intended for routine analysis and macroscopic evaluation, total nucleated cell count (TNCC), erythrocyte count, and morphological evaluation, was analyzed within 1 h of collection and the second aliquot was stored at -80 °C until 16S rRNA sequencing analysis.

### DNA extraction and quantification

DNA extraction from CSF was performed by using three different extraction kit, including: QI Amp® DNA Mini Kit (Qiagen GmbH, Hilden, Germany), Bacterial Genomic DNA Isolation Kit (Norgen Biotek Corp., Thorold, Canada) and DNAzol™ (Thermo Fisher Scientific, Waltham, USA). The DNAzol™ extraction protocol was the one that led to a suitable final DNA concentration for the Next Generation Sequencing (NGS) library production.

DNA concentration was determined using a NanoDrop 2000 spectrophotometer (Thermo Fisher Scientific).

### Library preparation, PCR enrichment and sequencing

In order to generate a suitable library, enrichment PCR reactions were performed towards the 16S rRNA V3–V4 hypervariable region, using the forward primer (5’ TCGTCGGCAGCGTCAGATGTGTATAAGAGACAGCCTACGGGNGCWGCAG) and the reverse primer (5’-GTCTCGTGGGCTCGGAGATGTGTATAAGAGACAGGACTACHVGGGTATCTAATCC). PCR Illumina standard conditions (16S Metagenomic Sequencing Library Preparation protocol, Illumina) were applied according to the manufacturer’s instructions for an initial analysis (first phase) and then modified (second phase) to achieve a more sensitive amplification as follows: 12.5 µL of 2x KAPA HiFi HotStart ReadyMix, 10 µL of each primer and 8 µL of 12.5 ng/µL of microbial DNA underwent initial denaturation at 95 °C for 3 min, then 25 cycles at 95 °C for 30 s, at 55 °C for 30 s, at 72 °C for 30 s, at 75 °C for 5 min and then maintained at 4 °C. Controls were added in each extraction and amplification step. Samples in which no genetic material was amplified were excluded from further analysis. The enriched 16S rRNA PCR library was then sent for normalization and quantification (Biomolecular Research Genomics) before proceeding with sequencing on an Illumina iSeq100 platform (Illumina) with a paired-end (PE) 2 × 300 bp protocol.

### Bioinformatic analysis

Reads were checked for sequence quality using FastQC (version 0.11.5) (Andrews 2010), trimmed with Trimmomatic software (release 0.39) (Bolger et al. 2014), and aligned with the bovine genome (Ensembl Bos_taurus ARS-UCD 1.2.101) using the BowTie2 (version 2.3.5.1) aligner (Langmead and Salzberg 2012) to eliminate possible host genomic material. The cleaned fastq files were then processed and analyzed using QIIME2 (Quantitative Insights Into Microbial Ecology, release 2020.8) (Bolyen et al. 2019). DADA2 (Callahan et al. 2016) (via q2-dada2 implementation) has been used to quality-filter and denoise sequence data: specifically, forward and reverse reads were further cleaned by trimming off the first ten positions and by truncating at positions 290 and 250, respectively, to remove low quality portions. The resulting amplicon sequence variants (ASVs), cleaned from chimeric sequences, were taxonomically classified by classify-sklearn command, as in q2-feature-classifier plugin, on the SILVA 138 Small SubUnit database using a 99% identity criterion [q2-silva-138-99-nb-classifier.qza]. A confidence level (CL) of ≥ 97% for identification of bacteria was set. The ASVs were further taxonomically characterized by NCBI BLAST (Basic Local Alignment Search Tool, release 2.11.0) by aligning them on the NCBI 16S bacterial database (2020_12_24). Only results with a single best hit and high similarity levels (> 98% of identical matches and eValue < 0.05) were considered. Phyla, classes, orders, families, and genera are reported as overall relative abundance, average relative abundance and standard error of the mean (SEM). Species are reported as overall relative abundance, in relation to ASVs identified at species level. Alpha diversities was evaluated by Shannon Index, Simpson Index, Faith’s Phylogenetic Diversity and Observed Feature measure and their significances were analyzed by Kruskal-Wallis test; beta diversity was calculated with Unweighted UniFrac and Normalized Weighted UniFrac distances, and with Bray-Curtis dissimilarity and their significances were analyzed by Permutational multivariate analysis of variance (PERMANOVA) test based on 1000 permutations. Rarefaction curves were produced to estimate species richness.

### Statistical analysis

Standard descriptive statistics are reported as median and interquartile range (IQR) for continuous variables and as percentage and frequency for categorical variables. Statistical analysis was performed using Excel (Microsoft, version 16.27) [19,071,500].

## Results

Ten animals were enrolled in this pilot study: 4 with a CNS infection and 6 with CNS disorders of other nature. The median DNA concentration in the CSF of the animals was 90 ng/µL (IQR 4.85–725).

Standard PCR yielded no genetic material from any of the samples. The modified 16S rRNA PCR amplification produced no bacterial genetic material in samples from 4 out of 10 patients: 1 with CNS infection-inflammation and 3 with a noninfectious inflammatory CNS disorder. These 4 were excluded from further analysis; the remaining 6 were: 4 males (66.67%) and 2 females (33.33%) of Piemontese (n = 3; 50%), Holstein Friesian (n = 1; 16.67%), Blonde d’Aquitaine (n = 1; 16.67%), and mixed breeds (n = 1; 16.67%), ranging from 3 days to 3 years of age (median: 2 months; IQR 13 days-6 months); the neuroanatomical localisation was: forebrain (n = 3; 50%), diffuse intracranial (n = 1; 16.67%), lumbosacral spinal cord segments (n = 1; 16.67%), and multifocal (n = 1; 16.67%).

The final diagnosis based on the VITAMIN D classification (Jaggy 2009) was: infectious-inflammatory disease (n = 3; 50%), metabolic/toxic (n = 2; 33.3%), and anomalous (1; 16.67%). Two groups were formed: an infectious-inflammatory group and a noninfectious inflammatory group.

In the infectious-inflammatory group, neonatal meningitis/meningoencephalitis(-myelitis) of suspected bacterial origin was diagnosed in 2 calves < 1 month that presented with 1–3 day history of neurological signs, which occurred following enteritis and omphalophlebitis in 1 calf (Case 1) and enteritis and arthritis in the other (Case 2). CSF analysis revealed mononuclear pleocytosis in both cases. One older calf (Case 3) (7 months old) presented with clinical signs consistent with meningitis/meningoencephalitis and 1–3 day history of seizures. CSF analysis showed marked mixed pleocytosis and increased total protein concentration. The patient was euthanized a few hours after the referral due to deterioration of neurological signs and onset of status epilepticus. Necropsy highlighted yellow-greenish thickening of the meninges at the level of the cerebellum, multifocal abscesses and mononuclear vasculitis at the level of the pons and cerebellum, edema, and inflammatory infiltrate in the brain parenchyma. All brain areas showed signs of predominantly mononuclear inflammation. The polymicrobial growth in the culture of brain samples was a mixed flora of slow-growing bacteria that in a standard culture system caused the failure to identify the single microorganisms. The noninfectious inflammatory group included 3 patients. A 3-month-old calf (Case 5) presented with central blindness and abnormal compulsive behaviour. CSF analysis was normal. Hypovitaminosis A was suspected; vitamin A therapy resolved all the clinical signs but not the blindness. A 3-year-old (Case 6) patient presented because of recurrent epileptic seizures. CSF analysis was normal and blood analysis revealed hypocalcemia. The patient died shortly after referral; necropsy was unremarkable. Hypocalcemia was the assumed cause of the neurological symptoms.

A 3-day-old calf (Case 7) presented with abnormal facial conformation and inability to stand on forelimbs and hindlimbs since birth. The diagnosis was complex congenital malformation of the head consistent with hydranencephaly or holoprosencephaly associated with other congenital facial abnormalities disclosed on post mortem autopsy (Di Muro et al. 2020).CSF analysis was normal.

CSF bacterial culture was performed in all 6 cases and resulted negative. Table 1 presents patients’ characteristics, amplification results and number of reads.

**Table 1:** Clinical Characteristics

Cases	Age (months)	Neuroanatomical localisation	Previous antibiotic therapy	TNCC/ µL	TP mg/dl	CSF interpretation	Diagnosis	Outcome	Amplification and reads number*
**Infectious-Inflammatory**									
**1**	1	Multifocal [ forebrain, spinal ´T3-L3)]	No	18.8	24	Mononuclear pleocytosis	NBSM	Died	Amplified (21,952)
**2**	0 (17 days)	Spinal (L4-S3)	Yes	21.7	32	Mononuclear pleocytosis	NBSM	Died	Amplified (35,586)
**3**	7	Forebrain	No	768	330	Mixed pleocytosis	BSM	Died	Amplified (9247)
**4**	0 (7 days)	Multifocal (forebrain, central vestibular)	Yes	205.6	232	Neutrophilic pleocytosis	NBSM	Died	Non amplified
**Non infectious-inflammatory**									
**5**	3	Forebrain	Yes	1.6	19	Normal	Hypovitaminosis A	Recovered	Amplified (33,293)
**6**	36	Forebrain	Yes	1	29	Normal	Hypocalcemia	Died	Amplified (33,305)
**7**	0 (3 days)	Diffuse intracranial	No	2	18	Normal	Holoprosencephaly/ Hydranencephaly	Died	Amplified (36,386)
**8**	24	Spinal (T3-L3)	No	1	26	Normal	Degenerative myelopathy	Died	Not amplified
**9**	72	Spinal (T3-L3)	No	1.4	25	Normal	Trauma	Recovered	Not amplified
**10**	12	Spinal (T3-L3)	No	1.2	18	Normal	Trauma	Recovered	Not amplified

TNCC denotes total nucleated cell count; TP: total protein; CSF: cerebrospinal fluid; NBSM: neonatal bacterial suppurative meningitis; BSM: bacterial suppurative meningitis

*Merged reads number is expressed in brackets

## 16S rRNA sequencing of DNA isolated from CSF

DADA2 denoising procedure, based on quality-based filtering, sequence inferring and merging, and chimera removal, led to 728 ASVs. Specifically, the median reads count per sample was 71750.5 (IQR 53,806–76,036); those obtained after elimination of host material, elimination of poor quality sequences, and pairing between forward and reverse sequences (indicated as merged) were about 70% of the starting counts (median per sample 49,699; IQR 37399.5–50,474). The sequences compared to the database were about 45% of the total as only the real sequences, not the chimeric ones, were considered (median per sample 33,299; IQR 24787.25–35015.75). The samples from Case 3 produced the lowest number of reads (total 20,344; merged 12,890; non-chimeric 9247), while the highest reads count was obtained from sample 5 (total 78,084; merged 50,650; non-chimeric 33,305).

**Fig. 1:**
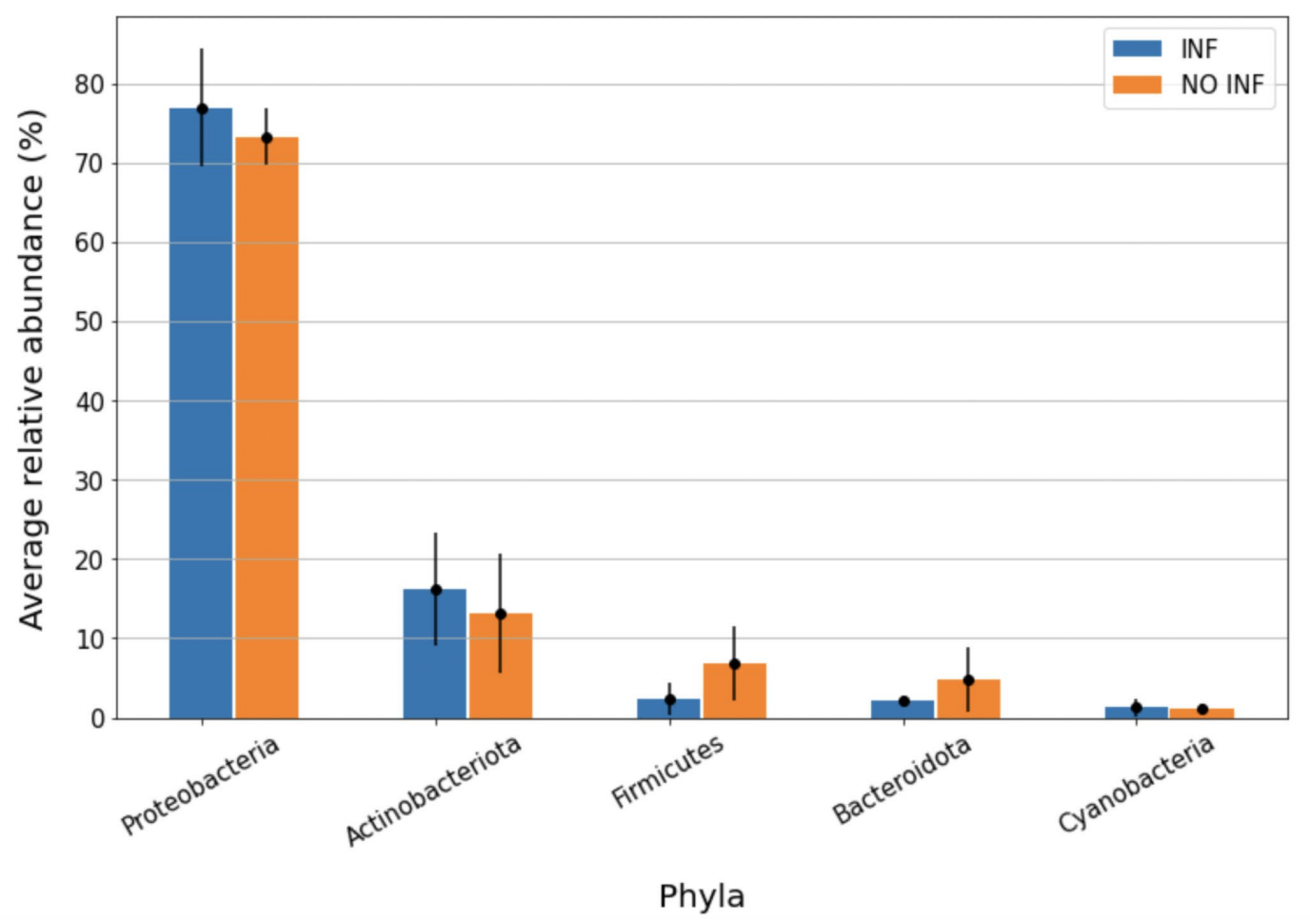
Average relative abundance of phyla in the infectious-inflammatory (INF) and non infectious-inflammatory (NON INF) samples. Only the 5 more present bacterial phyla were represented. Blue columns represent INF (n = 3) samples, while orange columns represent NON INF (n = 3). The bars represent the standard error of the mean

**Fig. 2:**
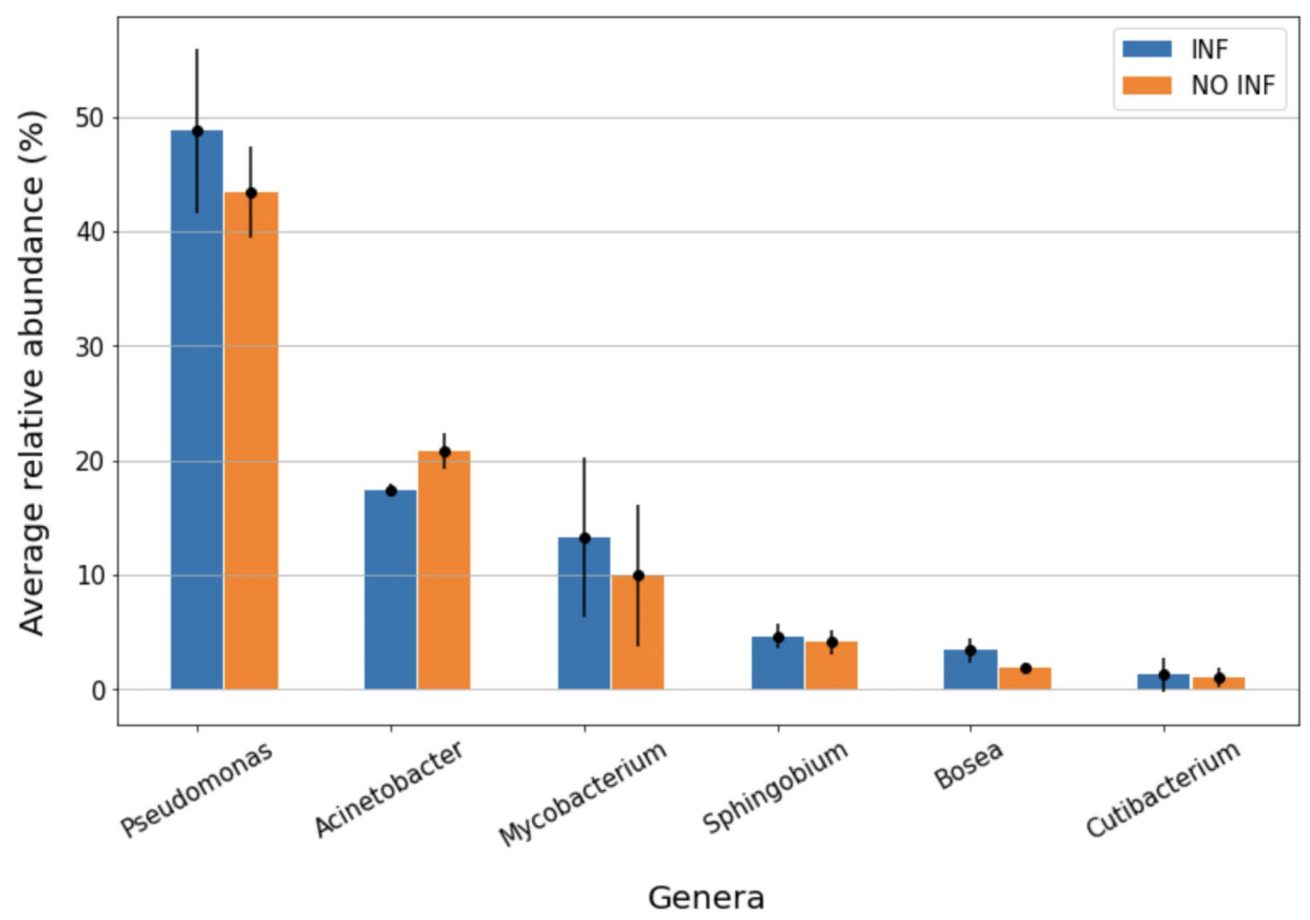
Average relative abundance of genera in the infectious-inflammatory (INF) and non infectious-inflammatory (NON INF) samples. Only the 6 more present bacterial genera were represented. Blue columns represent INF (n = 3) samples, while orange columns represent NON INF (n = 3). The bars represent the standard error of the mean

Each ASV resulting from DADA2 denoising step was then compared to the SILVA database to obtain a classification into a microbial taxonomy. A total of 17 phyla, 27 classes, 71 orders, 115 families and 150 genera were identified and the most present are reported in Table 2. Figures 1 and 2 illustrate phylum and genera abundance and composition in the infectious-inflammatory and non infectious-inflamamtory groups.

**Table 2:** Most present phyla, classes, orders, families and genera presented in descending order and reported as overall relative abundance, average relative abundance and standard error of the mean (SEM)

Phyla (n = 17*)	Classes (n = 27*)	Orders (n = 71*)	Families (n = 115*)	Genera (n = 150*)
Proteobacteria (73.56%; 75.07%, ± 2.27%)	Gammaproteobacteria (64.87%; 66.03%, ± 1.91%)	Pseudomonadales (64.12%; 65.19%; ± 2.03%)	Pseudomonadaceae (44.61%; 46.11%, ± 2.43%)	Pseudomonas (45.42%; 46.10%, ± 2.44%)
Actinobacteriota (15.25%; 14.72%, ± 2.76%)	Actinobacteria (15.06%; 14.55%, ± 2.82%)	Corynebacteriales (12.69%; 12.34%, ± 2.58%)	Moraxellaceae (19.54%; 19.08%, ± 0.86%)	Acinetobacter (19.91%; 19.08%, ± 0.86%)
Firmicutes (5.06%; 4,63%, ± 1.66%)	Alphaproteobacteria (8.70%; 9.04%, ± 0.69%)	Sphingomonadales (4.47%; 4.55%, ± 0.44%)	Mycobacteriaceae (11.80%; 227 11.59%, ± 2.50%)	Mycobacterium (12.01%; 11.59%, ± 2.51%)
Bacteroidota (3.81%; 3.48%, ± 1.21%)	Bacteroidia (3.82%; 3.48%, ± 1.21%)	Rhizobiales (3.56%; 3.84%, ± 0.55%)	Sphingomonadaceae (4.47%; 4.55%, ± 0.44%)	Sphingobium (4.35%; 4.36%, ± 0.41%)
Cyanobacteria (1.30%; 1.19%, ± 0.35%)	Bacilli (2.56%; 2.58%, ± 0.73)	Lactobacillales (1.85%; 1.99%, ± 0.59%)	Beijerinckiaceae (2.77%; 2.97%, ± 0.51%)	Bosea (2.56%; 2.68%, ± 0.43%)
	Clostridia (2.42%; 1.99%, ± 1.96%)	Flavobacteriales (1.71%; 1.64%, ± 0.34%)	Weeksellaceae (1.44%; 1.42%, ± 0.35%)	Cutibacterium (1.33%; 1.13%, ± 0.45%)
		Bacteroidales (1.54%; 1.31%; ± 1.03%)	Propionibacteriaceae (1.30%; 1.13%, ± 0.45%)	Chryseobacterium (1.32%; 1.30%, ± 0.38%)
		Propionibacteriales (1.32%; 1.15%, 225 ± 0.46%)	Streptococcaceae (1.12%; 1.40%, ± 0.60)	Streptococcus (1.08%; 1.35%, ± 0.60%)
		Oscillospirales (1.07%; 0.87%, ± 0.84%)		

*Numbers in brackets refer to the total number of phyla, classes, orders, families and genera identified

Only 11 out of 728 ASVs were identified at the species level, accounting for 1.51% of the ASVs and 0.69% of total abundance. The most abundant were: *Corynebacterium kroppenstedtii* (49.70%), *Lactobacillus algidus* (14.05%), *Pseudomonas pertucinogena* (8.50%), *Cutibacterium granulosum* (6.61%), *Bifidobacterium bifidum* (6.37%), *Treponem porcinum* (5.19%), *Corynebacterium maris* (3.07%), *Sphingobacterium jejuense* (2.71%), *Pseudomonas formosensis* (2.60%). *Negativicoccus succinicivorans* and *Peptoniphilus duerdenii* accounted for < 1% of the identified species.

Both alpha and beta diversity analyses did not detect any differences between the microbial communities of the two groups. Alpha and beta diversity analyses values are reported in Additional file 1. The rarefaction curves reached a plateau, indicating good representation of the microbial community. The rarefaction curve plots are reported in Additional File 2.

By blasting the ASVs versus the NCBI database, we assigned 169 ASVs to a species, 105 of which were ASVs previously recognized by QIIME with a CL > 97% and 64 ASVs with a CL < 97%. The former accounted for 14.28% of ASVs and 11.61% of total abundance, the most abundant identified species were: *Acinetobacter johnsonii* (43.46%), *Chryseobacterium profundimaris* (10.73%), *Pseudomonas helmanticensis* (4.94%), *Psychromonas aquatilis* (4.54%), *Afipia massiliensis* (4.50%), *Carnobacterium gallinarum* (2.92%), *Staphylococcus hominis* (2.39%), *Anaerococcus nagyae* (1.64%), *Acinetobacter indicus* (1.46%), *Brevundimonas naejangsanensis* (1.37%), *Rothia endophytica* (1.21%), and *Corynebacterium pilbarens* (1.06%). Six of the 11 ASVs assigned to a species by QIIME were likewise identified by BLAST and there was correspondence between them.

## Discussion

With the present study we evaluated for the first time the feasibility of 16S rRNA gene sequencing of DNA isolated from CSF of cattle with CNS disorders. Also, we evaluated the CSF microbiota composition in patients with CNS infection-inflammation and CNS disorders of other nature.

Despite the attempt to increase sensitivity by means of modified PCR, not all samples led to bacterial genetic material detection. Possible explanations are the very low CSF biomass, since the amount of CSF that can be collected is usually very small (a few mL) and the concentration of the genetic material in CSF is low (Moon et al. 2019). In addition, previous (before CSF collection) exposure to antimicrobials in 4 of the 10 patients could have reduced the bacterial mass even further. Other possible causes, like prolonged time between onset of symptoms and CSF collection (Ramachandran & Wilson 2020) and sample storage errors, were ruled out because CSF was collected shortly after the onset of clinical signs and the samples were correctly stored at -80 °C to prevent degradation of DNA and RNA.

By gene sequencing we detected microorganisms in the CSF samples from all six patients; however, our findings did not allow us to highlight any differences in the microbial population between the two groups.

Moreover, none of the more prevalent genera found were among those commonly encountered as cause of CNS infections in cattle at the present time. In other words, both groups, although they differed in their clinical features and diagnosis, presented similar microbial communities. To the authors opinion, there are different explanations of the presence of microorganisms in the CSF samples: sampling contamination or laboratory contamination, the presence of a CSF microbial community, and blood-CSF-barrier breakdown. A minimal external contamination during sample collection, despite all possible precautions taken to maintain sterility, cannot be ruled out as 16S rRNA sequencing of DNA isolated from samples from the environment was not performed. However, the microorganisms highlighted in this study do not reflect those typical of the skin microbiota of cattle **(**Bay et al. 2018). Laboratory contamination, which has been described in previous cases (Salter et al. 2014) was excluded by analysis of the reagent controls that was consistently negative. Alternatively, the bacteria identified could represent the microbiological community of the CSF in healthy cattle. Finally, given that the noninfectious inflammatory cases demonstrated neurological disorders, the blood-CSF-barrier might have been breached, resulting in contamination of the CSF from other body districts, as described in human medicine (Sweeney et al. 2018; Van Dyken and Lacoste 2018).

The results of the present study did not allow to differentiate the microorganism population between the two groups. Two options can be advanced to explain our results and the lack of pathogens identification in the infectious inflammatory group. First, classification did not reach the species level in most cases. Commensal and pathogenic bacterial species can share the same genus. For instance, the genus *Pseudomonas*, abundantly found in the samples from all patients, includes *Pseudomonas aeruginosa*, a common pathogen known to cause CNS infection in humans (Huang et al. 2007), and other species ubiquitous in the environment but not pathogenic (Gomila et al. 2015). Another possible explanation could be that the pathogens were not present in the CSF but rather in the nervous system parenchyma. It is possible that free pathogenic bacteria in CSF are transient and that they are present only in minimal quantities in this substrate. Unfortunately, 16S rRNA gene PCR and sequencing of DNA isolated from brain and spinal cord samples was not performed in the present study to confirm this hypothesis.

The issue of accurate taxonomic identification deserves consideration. The ability to identify bacteria with high taxonomic resolution depends in part on the sequencing technique. We sequenced only the V3-V4 hypervariable regions of the 16S gene (partial 16S sequencing). While it has been reported that the most variable regions are sufficient to identify genera, they are unlikely to adequately discriminate between species (Johnson et al. 2019). When we blasted the ASVs versus the GenBank NCBI database, more species were identified (1.15% of ASVs versus the SILVA database and 14.28% versus the NCBI database). The reason could be related to the different update status of these database: the SILVA database was last updated in 2019, whereas the GenBank NCBI is updated every two months, which could explain the greater ability of the latter to identify microorganisms at the species level.

*Acinetobacter johnsonii* was the most abundant species when we blasted the ASVs versus the NCBI database. This bacterium is a free-living, saprophytic organism that can be isolated from soil, water, sewage, and a wide variety of foods. *A. johnsoni* meningitis has been rarely reported in humans and it is usually seen in postneurosurgical patients with nosocomial infection (Chang et al. 2000). In the present study, *A. johnsonii* was equally found in both the infectious inflammatory and in the noninfectious inflammatory group and to the authors opinion it shouldn’t be considered a causative agent of CNS infection in cattle.

The present study has several limitations. The low number of cases included prevented us from achieving significant results. Four of the 10 cattle from the initial population were excluded, as no sufficient genetic material was obtained, although one patient was diagnosed with an infectious inflammatory CNS disease. However, despite the small sample size, this study presents the first description of the CSF microbiome composition of cattle. Further studies with larger sample size on microbiome analysis of CSF in cattle are warranted. In addition, previous exposure to antibiotics may have created a bias, but the use of antibiotics could not be prevented. A further limitation is the lack of CSF samples from healthy animals as controls. However, the procedure, although performed by skilled veterinarians, is potentially associated with risks for the patients. Furthermore, it requires pharmacological restrictions of the animals, raising ethical concerns in healthy subjects. On the other hand, post-mortem collection at the slaughter house is unrealistic because of the unpreventable contamination of the CSF by the captive-bolt stunning procedure. Our finding of a microbial community in the CSF of cattle with various neurological disorders is a valid starting point for future studies, with the inclusion of an ethical approved control group of healthy animals, that aim to validate this method as a diagnostic approach, reliable in clinical setting.

Finally, no etiological diagnosis was made in any of the infectious inflammatory cases. Bacterial cultures positive for one or more specific microorganisms could have aided in the interpretation of the NGS data. According to the literature, however, bacterial culture of CSF is only rarely positive (de Lahunta et al. 2021; Scott 2004). Other ways to confirm 16S rRNA sequencing findings, such as other NGS techniques, microarray analysis, PCR assay for specific pathogens or bacterial culture from brain tissue need to be investigated.

## Conclusions

We evaluated the applicability and utility of 16S rRNA sequencing for the study of CSF microbial community in cattle. Our results indicate that 16S rRNA of DNA isolated from CSF can lead to bacterial identification and taxonomic classification at the genus level. The findings of this pilot study are preliminary. Further studies, in which CSF samples from healthy animals and from the environment are included as controls, are needed in order to better evaluate CSF collection in the field. Positive culture or other innovative techniques for bacterial identification from nervous specimens could serve as positive controls. Our results indicate that a microbial community in the CSF independent of CNS infection cannot be ruled out.

## Supplementary information

**Additional file 1**: 

**Table S1** Alpha diversities calculated for the infectious inflammatory (INF) and non infectious inflammatory (NON INF) groups. Observed features, Shannon’s diversity index, Simpson index and Faith’s Phylogenetic Diversity values are reported as mean ± standard error and their corresponding P value.

**Table S2** Beta diversities calculated for the infectious inflammatory (INF) and non infectious inflammatory (NON INF) groups. Comparisons based on unweighted unifrac distance, weighted normalized unifrac distance and Bray-Curtis dissimilarity are reported through their pseudo-F ratio statistics and P Value.

**Additional file 2**: 

**Panel 1** Rarefaction curve showing the Shannon Alpha Diversity estimation as a function of sequencing depth for all the six analyzed subjects rarefaction curves plot.

**Panel 2** Rarefaction curve showing the Shannon Alpha Diversity estimation as a function of sequencing depth for the subjects grouped into the two classes.

**Additional file 3**: 

**Table S3** Table containing ASVs identifiers, taxonomic assignment, taxonomic assignment confidence level, ASVs abundance for the 6 subjects.

## Electronic supplementary material

11259_2022_9949_MOESM1_ESM.docx (PDF 217 kb)

11259_2022_9949_MOESM2_ESM.docx (PDF 333 kb)

11259_2022_9949_MOESM3_ESM.xlsx (PDF 61 kb)

## Data Availability

Datasets supporting the conclusions of this article are included within the article and its supplementary files. If necessary, further data generated during the current study can be available from the corresponding author on reasonable request.

## Abbreviations

CNS: Central nervous system.
CSF: Cerebrospinal fluid.
RNA: Ribonucleic acid.
PCR: Polymerase Chain Reaction.
TNCC: Total nucleated cell count.
DNA: deoxyribonucleic acid.
QIIME: Quantitative Insights Into Microbial Ecology.
ASVs: Amplicon sequence variants.
NCBI: National center for biotechnology information.
BLAST: Basic Local Alignment Search Tool.
TP: total protein.
NBSM: neonatal bacterial suppurative meningitis.
BSM: bacterial suppurative meningitis.
NGS: next generation sequencing.
