# Supplementary material for: Feasibility of 16S rRNA sequencing for cerebrospinal fluid microbiome analysis in cattle with neurological disorders: a pilot study
Source: Vet Res Commun. 2022 Jun 27;47(2):373–83. doi: 10.1007/s11259-022-09949-w (PMC10209220; doi:10.1007/s11259-022-09949-w)
Supplement: Supplementary file 1 — 11259_2022_9949_MOESM1_ESM.docx (PDF 217 kb) [file 11259_2022_9949_MOESM1_ESM.docx]

**Feasibility of** **16S rRNA sequencing for cerebrospinal fluid microbiome analysis in cattle with neurological disorders: a pilot study**

**Veterinary Research Communications**

Sara Ferrini^1^, Elena Grego^1^, Ugo Ala^1^, Giulia Cagnotti^1^*, Flaminia Valentini^1^, Giorgia Di Muro^1^, Barbara Iulini^2^, Maria Cristina Stella^1^, Claudio Bellino^1^, Antonio D’Angelo^1^

^1^Department of Veterinary Sciences, Clinical section, University of Turin, Largo Paolo Braccini 2, 10095 Grugliasco, TO, Italy; ^2^ Istituto Zooprofilattico del Piemonte Liguria e Valle d'Aosta, Turin, Italy

*Correspondence: [giulia.cagnotti@unito.it](mailto:giulia.cagnotti@unito.it)

**Additional file 1. Table S1**

|  | **INF (n= 3)** | **NON INF (n= 3)** | **P Value** |
| --- | --- | --- | --- |
| **Observed features** | 97.33 ± 22.34 | 216.33 ± 115.17 | 0.38 |
| **Shannon Index** | 4.10 ± 0.38 | 4.94 ± 0.52 | 0.28 |
| **Simpson Index** | 0.89 ± 0.02 | 0.92 ± 0.01 | 0.13 |
| **Faith’s Phylogenetic Diversity** | 18.54 ± 2.66 | 23.82 ± 8.92 | 0.51 |

**Additional file 1. Table S2**

|  | **Pseudo-F ratio** | **P Value** |
| --- | --- | --- |
| **Unweighted unifrac distance** | 1.12 | 0.31 |
| **Weighted normalized unifrac distance** | 0.74 | 0.83 |
| **Bray-Curtis dissimilarity** | 1.19 | 0.38 |
