# Supplementary material for: Feasibility of 16S rRNA sequencing for cerebrospinal fluid microbiome analysis in cattle with neurological disorders: a pilot study
Source: Vet Res Commun. 2022 Jun 27;47(2):373–83. doi: 10.1007/s11259-022-09949-w (PMC10209220; doi:10.1007/s11259-022-09949-w)

**Feasibility of** **16S rRNA sequencing for cerebrospinal fluid microbiome analysis in cattle with neurological disorders: a pilot study**

**Veterinary Research Communications**

Sara Ferrini^1^, Elena Grego^1^, Ugo Ala^1^, Giulia Cagnotti^1^*, Flaminia Valentini^1^, Giorgia Di Muro^1^, Barbara Iulini^2^, Maria Cristina Stella^1^, Claudio Bellino^1^, Antonio D’Angelo^1^

^1^Department of Veterinary Sciences, Clinical section, University of Turin, Largo Paolo Braccini 2, 10095 Grugliasco, TO, Italy; ^2^ Istituto Zooprofilattico del Piemonte Liguria e Valle d'Aosta, Turin, Italy

*Correspondence: [giulia.cagnotti@unito.it](mailto:giulia.cagnotti@unito.it)

**Additional file 2. Panel 1**


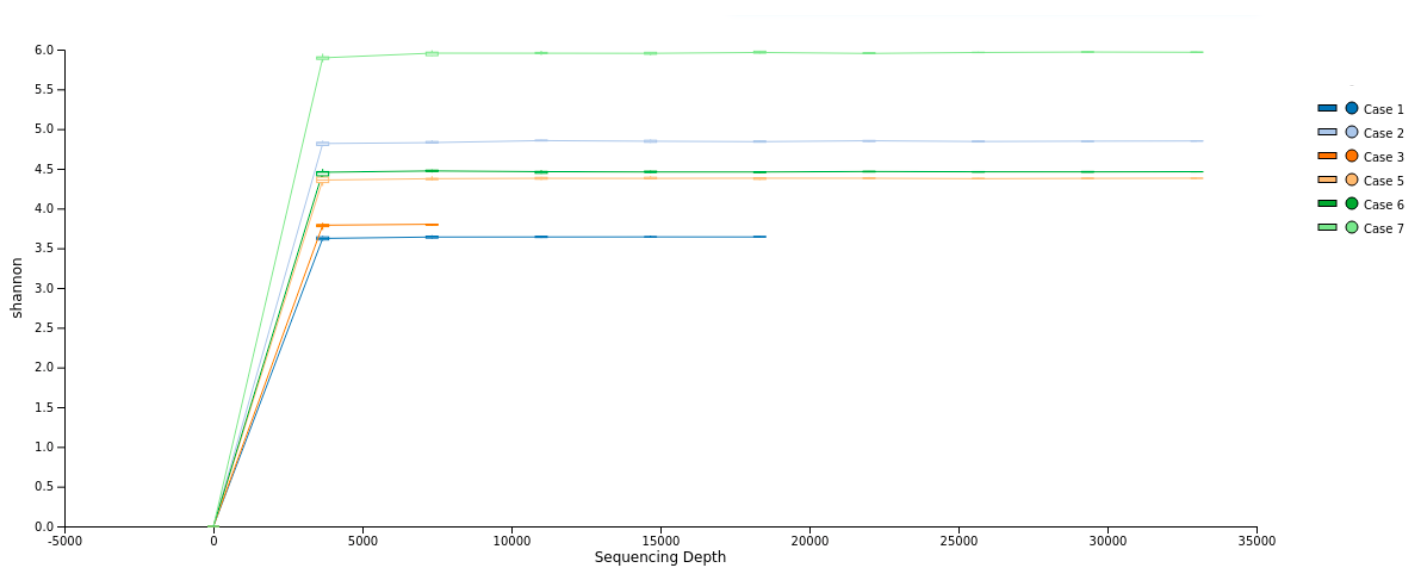



**Additional file 2. Panel 2**


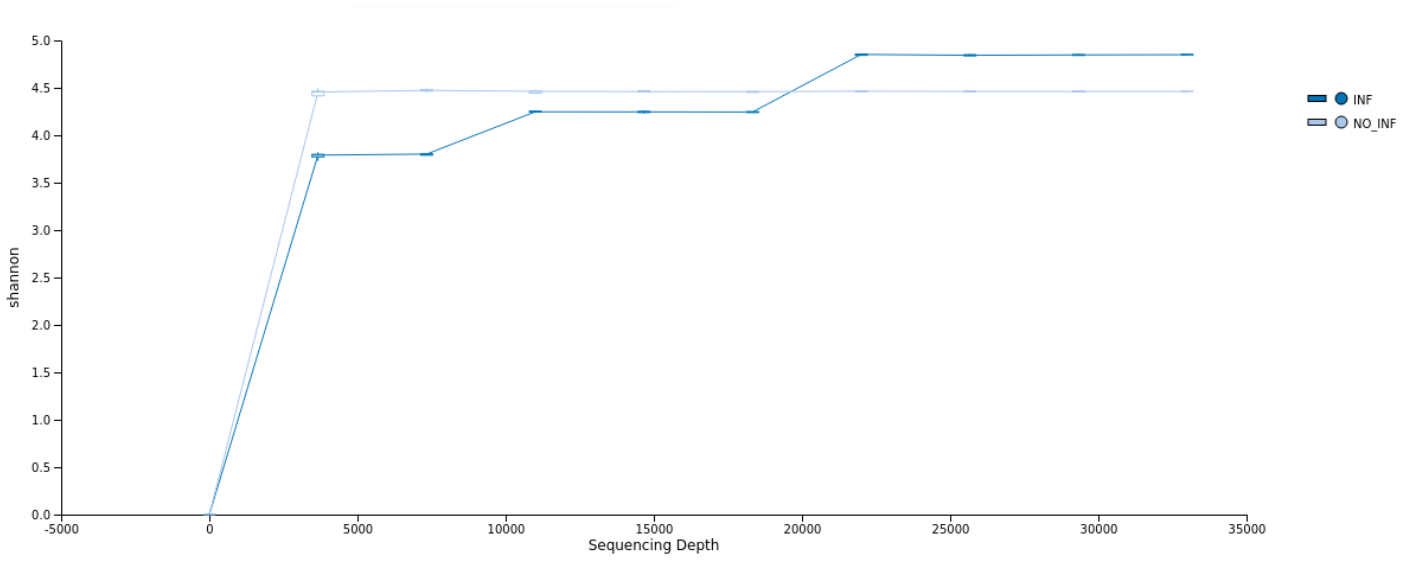


Supplement: Supplementary file 2 — 11259_2022_9949_MOESM2_ESM.docx (PDF 333 kb) [file 11259_2022_9949_MOESM2_ESM.docx]
